# Evaluation of biodentine pulpotomy in caries-exposed symptomatic vital mature permanent teeth in 9‒13-year-old children: A 24-month clinico-radiographic observation

**DOI:** 10.34172/joddd.2022.042

**Published:** 2022-12-30

**Authors:** Archana Singh, Harsimran Kaur, Priyanka Soni, Rishika Choudhary, Ramakrishna Yeluri

**Affiliations:** ^1^Department of Pedodontics & Preventive Dentistry, Runta College of Dental Sciences and Research, Chhatisgarh, India; ^2^Department of Pedodontics & Preventive Dentistry, Teerthanker Mahaveer Dental College & Research Centre, Uttar Pradesh, India; ^3^Department of Conservative and Endodontics, Buddha Institute of Dental Sciences and Hospital, Bihar, India

**Keywords:** Biodentine, Irreversible pulpitis, Pulpotomy

## Abstract

**Background.:**

The present study evaluated the clinical and radiographic outcomes of Biodentine pulpotomy for 24 months in symptomatic vital mature permanent teeth with caries exposure.

**Methods.:**

Seventy-three patients with a chief complaint of spontaneous pain in permanent teeth were screened. Finally, 47 mature permanent teeth underwent a Biodentine pulpotomy procedure. Clinical evaluation of 47 teeth was carried out at 1, 3, 6, 9, 12, and 24 months and radiographic evaluations were made at 6, 12, and 24 months. The success of Biodentine pulpotomy was evaluated using Pearson’s chi-square test. The significance level was determined at *P*<0.05.

**Results.:**

At 24 months, the clinical and radiographic success rate was 97.78%, with only one clinical failure at 9 months.

**Conclusion.:**

The clinical and radiographic success of Biodentine pulpotomy was high (97.78%). Thus, Biodentine pulpotomy can be an alternative to root canal treatment (RCT) in symptomatic vital mature permanent teeth.

## Introduction

 The dental pulp is one of the vital components of tooth structure that maintains the vascularization, nutrition, and support of the tooth. Loss of the dental pulp can lead to loss of tooth sensitivity, proprioception, and damping properties. Therefore, vital pulp treatment is undertaken to sustain the vitality of teeth, promote pulp tissue healing, facilitate the induction of reparative dentin and render the tooth asymptomatic.

The presence of spontaneous or severe preoperative pain does not always indicate that the pulp is incapable of repair, and deep carious lesions are not unconditionally related to an irreversible pattern of pulp pathology.^1^

Pulpotomy is a well-known procedure in deciduous teeth for pulpal inflammation limited to the coronal portion. For mature permanent teeth, even if the carious exposure is 1 mm^2^, conventional root canal treatment (RCT) is carried out. There is always controversy about performing pulpotomy on such permanent teeth. Pulpotomy in immature permanent teeth helps preserve radicular pulpal tissue for root formation.^2^ It is also considered an emergency procedure for permanent mature teeth until RCT can be accomplished.^3^

In child patients, for symptomatic vital mature permanent teeth, RCT is a complicated and prolonged process that often requires multiple appointments. However, pulpotomy in such teeth can be carried out in a single visit. Thus, it can be a substitute for RCT in children.^4^

For many years, calcium hydroxide (CH) has been used as a gold standard pulpotomy agent. Later, mineral trioxide aggregate (MTA) was suggested for its better properties. However, it has the potential for tooth discoloration, leading to the introduction of a new bioactive material, Biodentine.^5^

Biodentine has been introduced as a new bioactive calcium silicate cement, also known as a “dentin substitute.” It is available in separate powder and liquid forms or a capsule and pipette. The composition of the powder of Biodentine includes tricalcium silicate as the main core material, dicalcium silicate, calcium carbonate, and iron oxide. The radiopacifier used in the powder is zirconium oxide. Water is used as a reducing agent, calcium chloride as an accelerator in setting reaction, and a modified polycarboxylate as a superplasticizer as the component of the liquid. It stimulates growth factors that help form dentin by inducing odontoblasts. Calcium silicate sets in the presence of water. The hydrated calcium silicate (C-S-H) gel is known to be formed by hydration. CH is also formed in this chemical setting reaction. A precipitate that looks like hydroxyapatite is formed by the phosphate ions. The shorter setting time (9‒12 minutes) and improved handling properties are claimed by the manufacturer of Biodentine.^6^

 Due to the sparse literature available on Biodentine pulpotomy in symptomatic vital permanent teeth with a closed apex in children, the present study evaluated the clinical and radiographic success of Biodentine pulpotomy for 24 months.

## Methods

**Figure 1:**
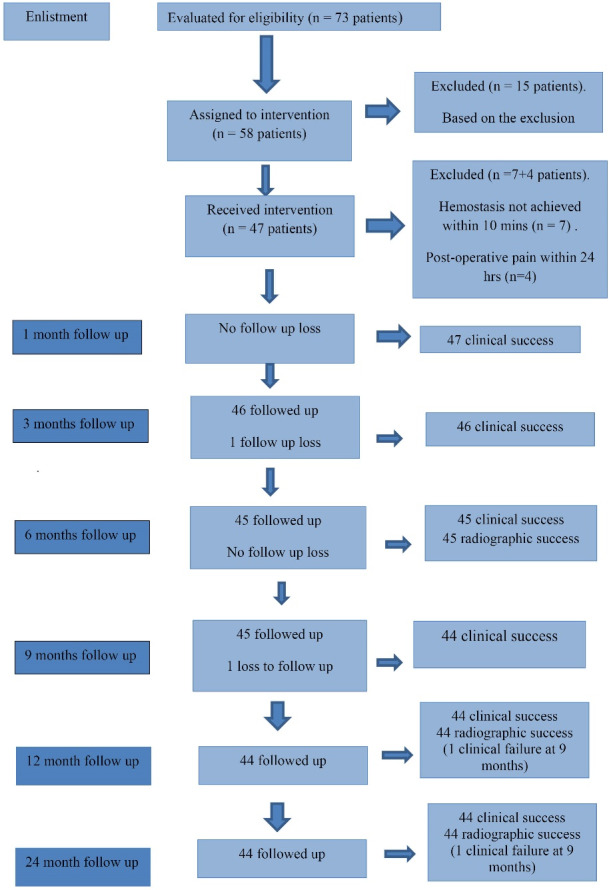
Flowchart of 73 eligible patients up to 24 months of follow-up

Seventy-three patients presenting with spontaneous pain were screened. Fifteen patients with non-vital, non-restorable teeth with periapical abscesses or sinus tracts, pathological mobility, internal or external resorption, and any root calcification associated with swelling were excluded. Fifty-eight carious but restorable teeth with pulpal exposure of more than 1 mm^2^, exhibiting symptoms of irreversible pulpitis and responding to pulp vitality test, were included in the study. Of 58 patients undergoing intervention, four reported postoperative pain within 24 hours, and in seven patients, hemostasis was not achieved within 10 minutes; therefore, 11 patients were excluded. Finally, 47 patients were eligible for observation at 1-, 3-, 6-, 9, 12, and 24-month intervals (Figure 1). Before starting the present study, the patients’ parents were provided with complete particulars regarding the study, the materials used, and their merits and demerits. The parents signed an informed consent form, allowing their children to participate in the study. The sample size was predetermined at 45 to estimate a success rate of 89.5% with 95% confidence and 10% relative precision. The sample size was adjusted to accommodate any dropouts at regular intervals. The sample size was determined using the following formula:

 N = Z2*P*Q/E2

 Z = normal table value (1.96)

 P = success rate, Q = 100-P

 E = precision

**Figure 2:**
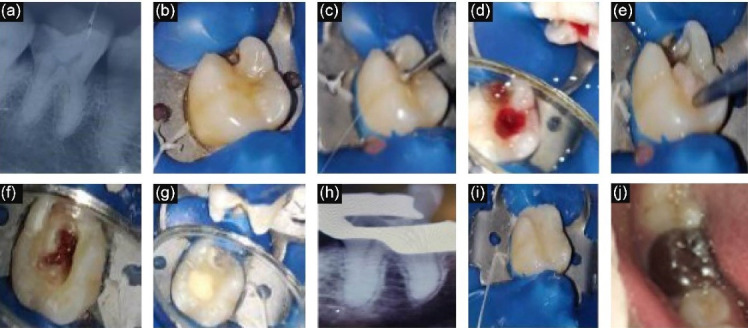
Clinical stages of pulpotomy. (a) Pre-op radiograph, (b) Tooth isolation, (c) Access cavity preparation, (d) Bleeding from canals, (e) Cotton pellet to achieve hemostasis, (f) Hemostasis achieved, (g) Placement of Biodentine, (h) Post biodentine radiograph, (i) GIC Restoration and (j) SSC placement

After applying local anesthesia (LA) (lignocaine with 2% adrenaline, W.I. Remedies Limited, India), isolation was carried out with a rubber dam. After complete extirpation of the coronal pulp, hemostasis was achieved using moist cotton pellets dipped in normal saline solution, and the time taken to achieve hemostasis was recorded. Only those cases where hemostasis was achieved within 5 minutes were included in the study, and the rest were excluded. Biodentine^TM^ (Septodont Inc., Saint-Maur-des-Fossés, France) was mixed as per the manufacturer’s instructions. A layer of Biodentine measuring 1.5‒2 mm in thickness was placed using an amalgam carrier and mildly packed with a condenser at the pulp chamber floor to cover the canal orifices. The position and the thickness of Biodentine were confirmed using radiovisiography. Then the material was allowed to set for 9‒12 minutes. The access cavity was sealed with type 2 glass ionomer restorative cement (GIC) (GC Gold Label, GC Corporation, Tokyo, Japan) (Figure 2). Twenty-four hours after the treatment, the patients were contacted by telephone to record any discomfort. In cases where postoperative pain was experienced within 24 hours, the respective tooth was treated with RCT and was excluded from the study. In cases where the postoperative period was uneventful, the teeth received stainless steel crowns (SSCs) (3MTM ESPETM Stainless Steel Primary Molar Crowns, 3M ESPA, Germany) as full coverage restoration in the subsequent appointment (Figure 2).

 All the included teeth were observed clinically at 1, 3, 6, 9, 12, and 24 months and radiographically at 6, 12, and 24 months. At each follow-up visit, each treated tooth was examined for the following adverse events: pain, swelling, sinus tract formation, tenderness on percussion or palpation, and radiographic evidence of peri-radicular or furcal pathology or root resorption. In cases of either clinical or radiographic failure, Biodentine pulpotomy was designated as a failure, and RCT was undertaken. The cases considered clinical or radiographic failure underwent RCT and were excluded from the study. Such teeth were considered a failure in clinical or radiographic and vice versa. The data obtained were subjected to statistical analyses using the chi-squared test.

## Results

The mean ages of males and females were 10.38 ± 1.22 and 10.40 ± 1.15 years, respectively. A total of 22 boys and 23 girls received the treatments and were followed clinically and radiographically for 24 months. Among the 47 patients that reported at the first follow-up of 1 month, there was 100% clinical success, and none reported pain, swelling, tenderness, mobility, and swelling. At the 3-month interval, one patient was lost to follow-up, and among the 46 (97.87%) reporting patients, there was 100% clinical success, with none of the patients reporting any symptoms. At the 6-month interval, one patient was lost to follow-up, and 45 (95.74%) patients reported no symptoms. At the 9-month interval, of 45 patients who attended the follow-up session, one patient developed pain while chewing, with tenderness on percussion. At the 9-month interval, clinical success was 97.78%. At the 12-month interval, 44 patients exhibited 97.78% clinical success. At the end of 24 months, the reporting 44 patients showed no clinical failure with 97.78% clinical success, as shown in Table 1.

**Table 1:** Clinical evaluation at follow-up time interval of 1, 3, 6, 9, 12 and 24 months

	**Absence of pain**	**Absence of tenderness**	**Absence of mobility**	**Absence of swelling**	**Absence of sinus**	**Success**
1 Month (n = 47)	Absent	Absent	Absent	Absent	Absent	100%
3 Months (1 lost to follow up) (n = 46)	Absent	Absent	Absent	Absent	Absent	100%
6 Months (1 lost to follow up) (n = 45)	Absent	Absent	Absent	Absent	Absent	100%
9 Months (1clinical failure) (n = 45)	Pain present in 1 tooth	Tender to percussion present in 1 tooth	Absent	Absent	Absent	100%
12 Months (1 clinical failure at 9 months) (n = 44)	Absent	Absent	Absent	Absent	Absent	97.78%
24 Months (1 clinical failure at 9 months) (n = 44)	Absent	Absent	Absent	Absent	Absent	97.78%
Chi-square test	0.924	0.924	0.924	0.924	0.924	
*P *value	0.903 (NS)	0.903 (NS)	0.903 (NS)	0.903 (NS)	0.903 (NS)	

NS, Non-significant.

**Figure 3:**
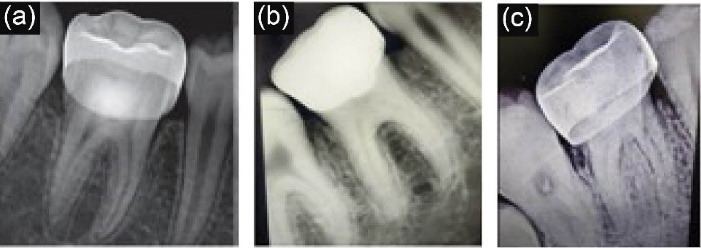
(a) 6 months follow up radiograph, (b) 12 months follow up radiograph and (c) 24 months follow up radiograph

Radiographic assessments were carried out at 6-, 12-, and 24-month intervals (Figure 3). At the 6-month recall, of the 45 patients, there was 100% radiographic success in terms of the integrity of lamina dura, furcal radiolucency, internal root resorption, external root resorption, and root canal obliteration. At the 12-month interval, of the 44 reporting patients, the success rate was 97.78% since there was one clinical failure at 9 months. Similar results were seen at the end of 24 months, as shown in Table 2.

**Table 2:** Radiographic evaluation at follow-up time interval of 6 and 12 and 24 months

	**Integrity of lamina dura**	**Furcal radiolucency**	**Internal root resorption**	**External root resorption**	**Canal obliteration**	**Success**
6 Months (1 teeth at 3 months and 6 months lost to follow up respectively) (n = 45)	Absent	Absent	Absent	Absent	Absent	100%
12 Months (1 clinical failure at 9 months) (n = 44)	Absent	Absent	Absent	Absent	Absent	97.78%
24 Months (1 clinical failure at 9 months) (n = 44)	Absent	Absent	Absent	Absent	Absent	97.78%
Chi-square test	0.344	0.344	0.344	0.344	0.344	
*P* value	0.557 (NS)	0.557 (NS)	0.557 (NS)	0.557 (NS)	0.557 (NS)	

NS, Non-significant.

## Discussion

The dental pulp has good potential to repair than regenerate. Depending on the immune status of the pulp, inflammation can be restricted to the pulp chamber, sparing the pulp in the root portion of teeth.^7^

Pulpotomy is indicated for caries-exposed permanent teeth in which root formation is not complete to help in the maturation of the root and lay down hard tissue at the site of pulp amputation. For mature permanent teeth, even if the carious exposure is 1 mm^2^, conventional RCT is undertaken. However, there has always been controversy about performing pulpotomy on such permanent teeth. Nevertheless, RCT is a complicated and prolonged process with several appointments for child patients with symptomatic vital mature permanent teeth. Also, full-coverage restorations after RCT make the treatment costly. However, in pulpotomy of teeth with irreversible pulpitis, with complete root formation, the pulp which is infected is only removed to set a favorable environment for the healing of pulp tissue. Chueh and Chiang^8^ carried out a study for the histological examination of permanent teeth with complete root formation and gross carious exposure with irreversible pulpitis. These teeth were histologically similar to the findings of pulpotomy in cases of reversible pulpitis in permanent teeth with open apex when treated with MTA pulpotomy.

According to Cvek,^9^ pulpotomy can be performed on mature permanent teeth in young patients because of the high healing capacity of pulp tissues. According to Matsuzaka et al,^10^ there is a capability to form dentin in the cells of pulp, but the rate of dentin formation diminishes with age.

The earliest material used for pulpotomy is CH, with the capacity to form tertiary dentin. The high pH after mixing CH with water makes dentin formation possible. Torabinejad et al^11^ advocated using tricalcium silicate-based inorganic medicament (MTA), which was more biocompatible and bioactive, for vital pulp treatment. Nevertheless, the significant demerits of MTA include staining of teeth, extended setting time, difficulty in handling, and high cost. These limitations of MTA have been addressed by the new calcium-silicate cement Biodentine (Septodont Inc., Saint-Maur-des-Fossés, France).

 Therefore, this study was undertaken to clinically and radiographically observe Biodentine pulpotomy in symptomatic vital permanent teeth with closed apices in children 9‒13 for 24 months.

There was 100% clinical success and no follow-up loss by the end of 1 month. At the end of 3 months, there was one follow-up loss, but clinical success was 100%. At the end of 6 months, there was also one follow-up loss with 100% clinical and radiographic success (*P* > 0.05). The high success rate of full pulpotomy can be attributed to full-coverage restorations by SSCs that formed the complete coronal seal. Similarly, Demarco et al^12^ found a statistically significant positive correlation between the features of restorations and the success of pulpotomy clinically in their retrospective study. It is also well-established that good-quality restorations in the coronal portion are vital for successful treatment in RCT.^13^ The microleakage through a faulty coronal restoration can degenerate the pulp tissue gradually.^14,15^ In contrast to the present study, Tan et al^16^ reported that most of the pulpotomised teeth had late failures due to the lack of coronal seal.

One clinical failure was noted at the end of 9 months, with a success rate of 97.78%. The patient had pain chewing, and the tooth was tender to percussion. RCT was successfully accomplished in the respective tooth. The failure may be ascribed to some late reactions shown by the pulp in response to Biodentine. Further histological examination of the pulp tissue is required to ascertain the reaction or biocompatibility of Biodentine with pulp tissue. At 12 and 24 months, the radiographic success was 97.78% due to one clinical failure at 9 months. Radiographic evaluations were carried out at 6-, 12-, and 24-month intervals to minimize radiation exposure in child patients, as suggested by the American Academy of Pediatric Dentistry (AAPD).^17^

In this study, no attempt was made to test the pulp vitality as complete pulpotomy was carried out, and final restoration was accomplished with SSC. Therefore, there was no scope for the pulp to respond to any pulp vitality test. Barngkgei et al^3^ performed partial pulpotomy with MTA cement in symptomatic permanent teeth and found that the electric pulp test showed delayed positive response of all tested teeth in all the follow-up visits; however, the test could not be performed on two teeth that had been crowned.

Additionally, due to full-coverage crown restorations, dentin bridge formation could not be recorded radiographically. The radiopacity of the SSC superimposed the radiopacity of Biodentine at the cervical junction. The failure of a tight coronal seal due to the plastic restorative materials leads to the failure of pulpotomy.^16^ Therefore, maintaining the teeth in a healthy state rather than evaluating dentine bridge formation was the priority of this study.

The present study reported a high percentage of clinical and radiographic success (97.78%), consistent with a study by Taha et al,^1^ where a clinical success rate of 100% and a radiographic success of 93.8% in one-year follow-up were found.

There are limited studies to depict the prognosis of pulpotomy in caries-exposed pulps in young molars. Barngkgei et al^3^ assessed the outcomes of pulpotomy treatments with MTA in symptomatic mature permanent teeth with caries exposures in adults with a mean age of 29 clinically and radiographically, and 100% success was reported. The sample size in their study was small; in contrast, this study included very young patients with a mean age of 10.36 years and a sample size of 45 molar teeth, which showed a success rate of 97.78%.

Taha and Abdelkhader^18^ reported a clinical success rate of 100% and a radiographic success rate of 93.8% in a one-year follow-up. For the irremediable pulpal inflammation in permanent teeth with closed apices, Biodentine pulpotomy procedures were performed in their study. The mean age of the sample was 33.2 years. This study showed similar results, with 97.78% clinical and radiographic success. Tan et al^16^ performed complete pulpotomy procedures using tricalcium silicate cement. Fifty-one patients with a mean age of 40 years were included. They found a success rate of 90.2%. The final restoration was accomplished with plastic restorative materials. They suggested that the cause of failure was inadequate sealing with bioactive material and coronal restoration. In contrast, the present study exhibited a high success rate (97.78%) due to the adequate coronal seal obtained using SSC.

Oginni and Udoye^19^ demonstrated that when RCT was performed in a single appointment, 18% of individuals returned with pain after one month. Asgary and Eghbal^20^ compared the mean pain intensity after RCT in one appointment and the pulpotomy procedure in cases of irreversible pulpitis in permanent teeth. The pulpotomy group showed substantially less pain than the samples in the RCT group after seven days. In this study, the pain diminished considerably after pulpotomy because of decreased local pressure, assembly of chemokines, and cutting of nociceptive sensory neurons.

###  Limitations od the study

 (1) short duration of follow-up; (2) absence of histological examination to ascertain the reaction and biocompatibility of Biodentine; (3) failure to perform vital pulp testing. Long-term clinical trials are advocated to confirm the benefits and prudency of Biodentine pulpotomy.

## Conclusion

 The gross success observed in full pulpotomy of mature permanent teeth was 97.78% in children between 9‒13 years by the end of 24 months. Biodentine pulpotomy can serve as a substitute for RCT in children to preserve the vitality of the radicular pulp. Future studies on pulpotomy of permanent mature teeth with a larger number of participants and prolonged follow-up durations are recommended. Also, histological studies can be beneficial in confirming the same. Furthermore, depending on the extent of infection/inflammation, a partial pulpotomy procedure can also be suggested in permanent mature teeth.

## Funding

 No funding resources.

## Ethics Approval

 The study was approved by the institutional ethics committee in the Teerthanker Mahaveer Dental College and Research Centre, Uttar Pradesh, India (Ethical approval no.: TMDCRC/IEC/PPD).

## Competing Interests

 No conflicts of interest.
